# Quantitative evaluation of myocardial layer-specific strain using two-dimensional speckle tracking echocardiography among young adults with essential hypertension in China

**DOI:** 10.1097/MD.0000000000012448

**Published:** 2018-09-28

**Authors:** Liangjie Xu, Ning Wang, Xinxin Chen, Yi Liang, Hong Zhou, Jinchuan Yan

**Affiliations:** a Department of Cardiology, Affiliated Hospital of Jiangsu University; b School of Medicine, Jiangsu University, Zhenjiang, Jiangsu Province, China.

**Keywords:** echocardiography, hypertension, myocardial layer-specific strain, young adults

## Abstract

The myocardial wall of the left ventricle is a complex, multilayered structure, which is altered in young adults with hypertension. The aim of this study was to define the characteristics of longitudinal and circumferential strain in young adults with hypertension.

Two-dimensional speckle tracking echocardiography was used to analyze longitudinal and circumferential strain parameters in 67 young adults with hypertension, 70 older young adults with essential hypertension and 62 healthy adults.

The global longitudinal strain (GLS) and global circumferential strain (GCS) was the highest at endocardium, and lowest at epicardium. A layer-specific analysis of myocardial deformation in all adults revealed that all of the peak systolic longitudinal strain (LS) and the peak systolic circumferential strain (CS) in the endocardium, mid-myocardium and epicardium were gradually increased from the base to the apex. The peak systolic LS showed significant differences at basal, mid-ventricular, and apical level among normal adults, young NLVH (nonleft ventricular hypertrophy), and young LVH (left ventricular hypertrophy). In all the adults with hypertension, young adults were associated with higher peak systolic longitudinal strain values compared with older adults, but the small differences of LS may be meaningless in clinical settings. Between the young LVH and older LVH, the peak systolic CS showed significant differences except data of epicardium at basal and mid-ventricular level.

This study provides reference values for layer-specific strain in young adults with hypertension. This detailed strain analysis provides layer-oriented information to reveal the different characteristics of circumferential and longitudinal strain in young adults with hypertension. This systolic dysfunction could be detected conveniently and accurately by 2DSTE.

## 1 Introduction

In China, young adults (18–39 year-olds) with hypertension have the lowest control rates among hypertensive adults. High blood pressure impairs the compliance of heart, and detrimentally affects left ventricular (LV) diastolic function. Uncontrolled hypertension also increases the risk of future cardiovascular event and the development of chronic kidney disease.^[1–4]^ Therefore, identification and understanding cardiac structure and function is of clinical importance and is essential for the management of young adults with hypertension.

Reduction of myocardial deformation accompanied with progressive cardiac hypertrophy according to the long exposure to high blood pressures in patients has been described in previous studies.^[5]^ These reports only shows the structure and function changes among patients in all ages. Previous studies mainly focused on the global myocardial wall thickness form endocardium to epicardium. However, the myocardial wall of the LV is a complex, multilayered structure and is not homogenous.^[6]^ The contribution of the different layers of myocardium to myocardial deformation in young adults with hypertension remains unknown. Recent advancements in 2D strain software have enabled the quantification of myocardial function in 3 layers.^[7–11]^ In this study, we used the advanced two-dimensional speckle tracking echocardiography (2DSTE) to evaluate myocardial deformation within the endocardial, mid-myocardial, and epicardial layer. The aim of this study was to define the alteration of longitudinal and circumferential strain in young adults with hypertension.

## 2 Methods

### 2.1 Study population

According to 2007 Guidelines for the management of arterial hypertension,^[12]^ hypertension was defined as systolic blood pressure ≥140 mm Hg or diastolic blood pressure ≥90 mm Hg. Sixty-seven young adults with essential hypertension (42 men, range, 18–39 year-olds), 70 older young adults with essential hypertension (47 men, range, 40–73 year-olds) and 62 age and sex matched, healthy adults were enrolled with informed consent. Patients with established coronary artery disease, echocardiographic evidence of either regional or global wall motion abnormalities, valvular heart disease, diabetes mellitus, renal disease, and hypertrophic cardiomyopathy were excluded.

According to LV mass index,^[13]^ all the adults with hypertension were divided into 2 group. A LV mass index >125 g/m^2^ in men and a LV mass index >110 g/m^2^ in women were considered to have LV hypertrophy (LVH). The patients without LVH were considered as NLVH.

### 2.2 Conventional two-dimensional Doppler echocardiography

Subjects underwent standard 2D echocardiographic examinations using commercially available ultrasound machine (Vivid E9; GE Healthcare, Horten, Norway) equipped with a M5S transducer. Three (basal, middle, and apical) of LV short axis views and 3 LV apical views (4-chamber, 2-chamber, and long-axis views) were acquire in the left lateral decubitus position during a breath hold. The following parasternal long-axis view as recommended^[14]^: interventricular septum (IVSD), posterior wall (LVPWD), left ventricular end-diastolic (LVEDs) diameters. LV ejection fraction and stroke volume were calculated as previously described.^[15]^ Relative wall thickness (RWT) was calculated as (IVSD + PWD)/LVEDd. LV mass was calculated using the formula proposed by Devereux et al and corrected by body surface area (BSA) to derive the LV mass Index.^[16]^ LV end-diastolic and end-systolic volumes and LVEF were calculated using biplane disk-summation algorithm,^[17]^ and the indexed by BSA. Pulse-wave Doppler examination of LV inflow and outflow and tissue Doppler examination at the mitral annulus was performed according to the ASE recommendations.^[18]^ Datasets were digitally stored on a hard disk for offline analysis.

### 2.3 2D speckle tracking echocardiography

**Figure 1:**
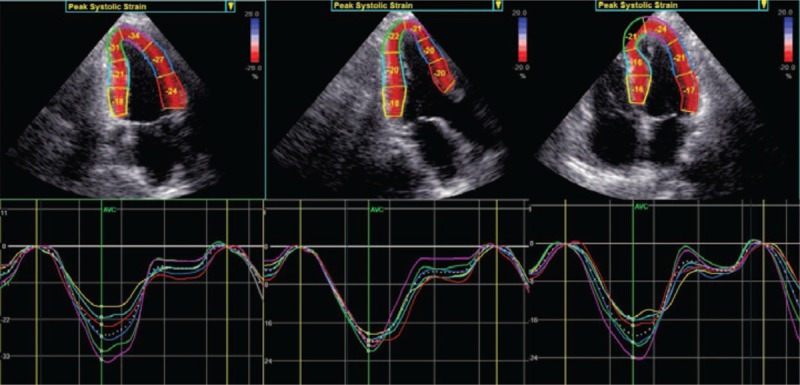
Layer-specific strain curves in each segment. Quantitative myocardial parameters for each segment are evaluated in an 18 segment LV model (6 segments at each level) at all three acquires parasternal long-axis views.

**Figure 2:**
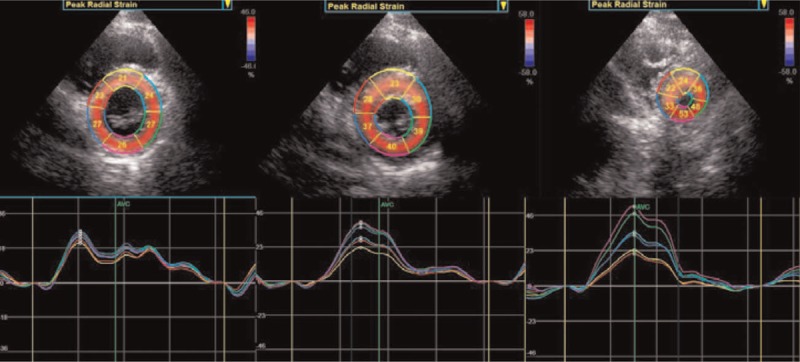
Layer-specific strain curves in each segment. Quantitative myocardial parameters for each segment are evaluated in an 18 segment LV model (6 segments at each level) at all 3 acquires parasternal short-axis views.

After acquired the apical long axis, 4- and 2-chamber views, parasternal short axis at the basal, middle, and apical levels of 3 consecutive cardiac cycles, the different views were analyzed using two-dimensional STE software (2D-Strain, EchoPac PC, version 113.0.5, GE Healthcare). We used the button apical long axis, apical 2 chamber, apical 4 chamber, short axis at the mitral valve level, short axis at the papillary level, and short axis at the apical level to sketch the subendocardial, respectively, and confirmed the aortic valve closure time in the apical long axis view, then the 2DSTE software was used to create a region of interest automatically, which contained subendocardial, middle, and subepicardical, adjusted the interest to make the myocardial included well. The software performed a speckle tracking analysis on the LV myocardium in each view. Upon delineating the region of interest, the software automatically generated time-domain strain curves in 6 segments with which end-systolic strain was subsequently calculated. GL(C)S was defined as the average longitudinal (circumferential) strain at the end-systole in 18 segments. Finally, the peak systolic longitudinal strain, circumferential strain of the subendocardial, middle, and subepicardial myocardial layers of the LV were calculated (Figs. 1 and 2).

### 2.4 Intraobserver and interobserver agreement

All imaging data were analyzed by one observer in random order. To test intraobserver variability, a single observer analyzed the data twice on occasions separated by an interval of 1 month. To test interobserver variability, a second observer analyzed the data without knowledge of the measurements of the first observer.

### 2.5 Statistics

Descriptive data are shown as means ± SD. GLS, GCS, LS, and CS were presented in their absolute value. Comparison of continuous variables was performed with independent sample *t* tests or analysis of variance (ANOVA) as appropriate. Reproducibility was assessed by the mean percentage error (absolute difference divided by the mean of the 2 observations). *P* < .05 was considered to indicate statistical significance.

## 3 Results

### 3.1 Clinical characteristics and echocardiographic variables

Clinical and echocardiography data from patients with hypertension and controls are shown in Table 1. No significant differences were found among all the patients in terms of BSA, BMI, or heart rate. There were significant differences in the left atrial diameter, thickness of the IVSD at end diastole, thickness of the posterior left ventricular wall at end diastole, E/A, and A among the patients with hypertension (young NLVH or LVH), and control group (*P* < .05; Table 1). The pattern in control adults, young NLVH, and young LVH adults in the left atrial diameter, thickness of the IVSD at end diastole were: young LVH > young NLVH > controls. The A value was minimum and the E/A was maximal in controls. The A value and the E/A of older NLVH were significantly lower than young NLVH. The E/A was much lower in older LVH compared with young LVH. There were no differences in E, or LV end-diastolic volume, end-systolic volume, or ejection fraction (*P* > .05; Table 1).

**Table 1:**
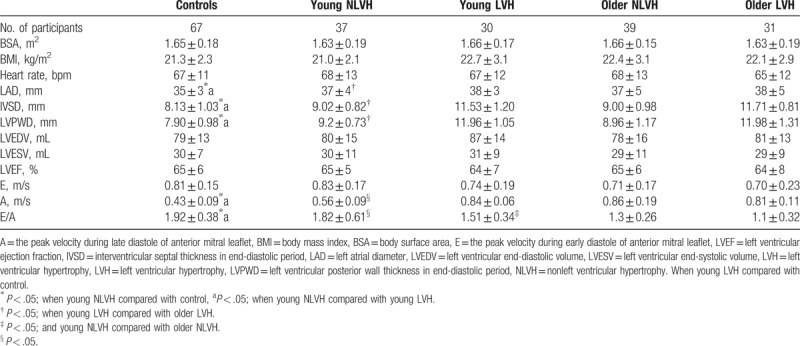
The basic information from conventional two-dimensional Doppler echocardiography.

### 3.2 Layer-specific longitudinal strain

**Figure 3:**
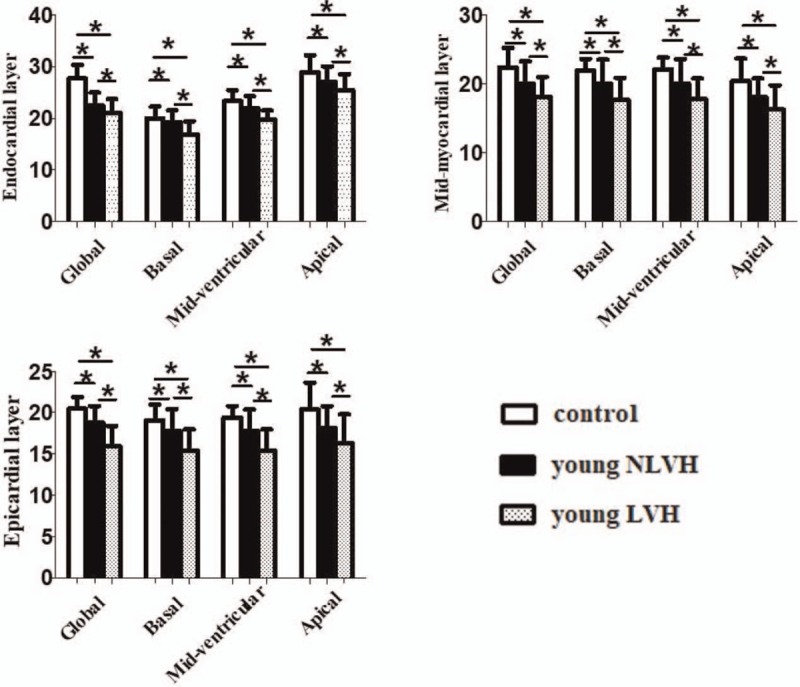
Three-layer longitudinal strain at the basal, mid-ventricular, and apical levels of the left ventricle between young adults with hypertension and normal adults. ^∗^*P* < .05.

The layer-specific GLS and peak LS at the basal, middle, and apical levels of the LV among the 5 groups classified according to LV mass index and age decade are provided in Table 2 and Fig. 3. Regarding global longitudinal strain, endocardial GLS was the highest, and the lowest at epicardium. GLS was markedly attenuated at endocardium, mid-myocardium, and epicardium in young LVH adults when compared with normal adults. GLS was markedly attenuated at endocardium, mid-myocardium, and epicardium in young LVH adults when young NLVH compared with young LVH. In all the adults with hypertension, there was no significant difference.

**Table 2:**
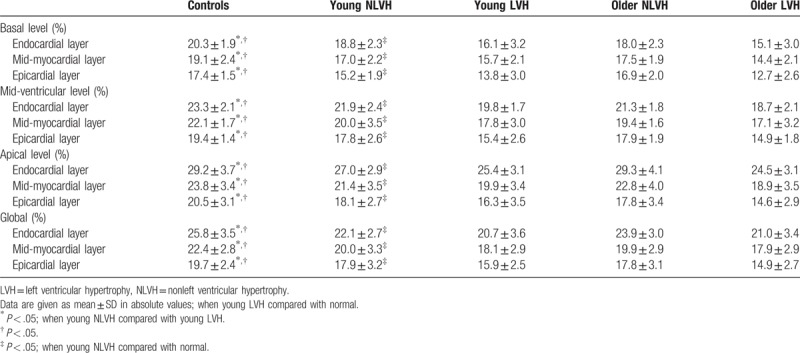
Three-layer longitudinal strain at the basal, mid-ventricular, and apical levels of the left ventricle in young adults with hypertension.

A layer-specific analysis of myocardial deformation in all adults reveal that all of the peak systolic LS in the endocardium, mid-myocardium, and epicardium were gradually increased from the base to the apex. When young adults with hypertension and normal adults were compared, all of the peak systolic LS had significant difference exclude the values detected at the basal level, the strain was normal adults > young NLVH > young LVH. When young NLVH was compared with young LVH, the peak systolic LS was markedly attenuated at the basal, mid-ventricular, and apical level. In all the adults with hypertension, young adults was associated with higher peak systolic longitudinal strain values compared with older adults, but the small differences of LS may be meaningless in clinical settings.

### 3.3 Layer-specific circumferential strain

**Figure 4:**
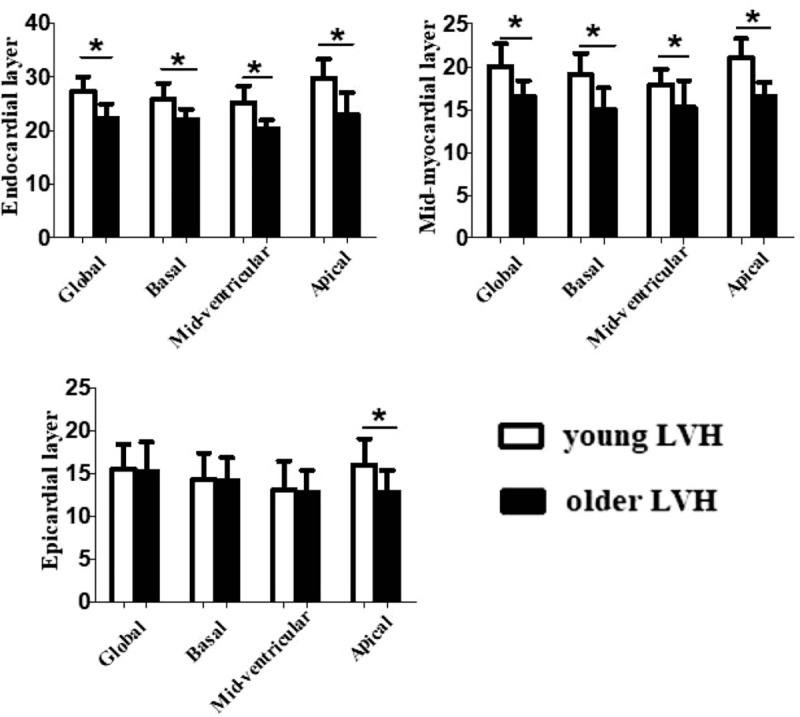
Three-layer circumferential strain at the basal, mid-ventricular, and apical levels of the left ventricle in all the adults with left ventricular hypertrophy in hypertension. ^∗^*P* < .05.

The layer-specific GCS and peak CS at the basal, middle, and apical levels of the LV among the 5 groups classified according to LV mass index and age decade are provided in Table 3 and Fig. 4. Similar to layer-specific GLS, endocardial GCS was the highest, and the lowest at epicardium. In all the adults with hypertension, the GCS had significant difference between young LVH and older LVH at endocardium and mid-myocardium.

**Table 3:**
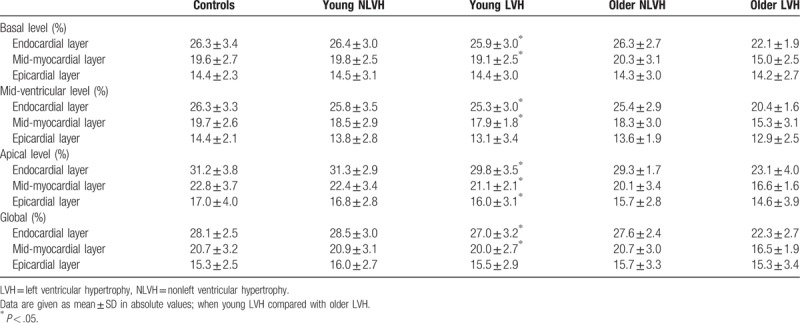
Comparison of the peak systolic circumferential strain of the subendocardial, the middle, and the subepicardial myocardial.

A layer-specific analysis of myocardial deformation in all adults revealed that all of the peak systolic CS in the endocardium, mid-myocardium, and epicardium were gradually increased from the base to the apex. In all the adults with hypertension, the peak systolic circumferential strain had significant difference except at epicardium of basal and mid-ventricular level between the young LVH and older LVH.

### 3.4 Observer variability

Intra- and interobserver variability for layer-specific GLS AND GCS are described in Table 4. Observer variabilities were lower for GLS measurements than those for GCS measurements. However, % variability of GCS was still less than 7%.

**Table 4:**
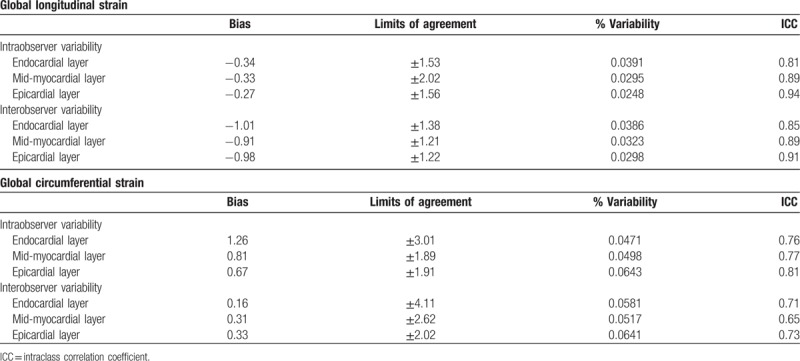
Intra- and interobserver variability in layer-specific global strain.

## 4 Discussion

The primary findings of this study are summarized as follows:

1. Regarding GLS and GCS, value of endocardial was the highest, and the lowest at epicardium. A layer-specific analysis of myocardial deformation in all adults revealed that all of the peak systolic LS and the peak systolic CS in the endocardium, mid-myocardium, and epicardium were gradually increased from the base to the apex.
2. Among normal adults, young NLVH, and young LVH, the peak systolic LS showed significant differences at basal, mid-ventricular, and apical level.
3. In all the adults with hypertension, young adults was associated with higher peak systolic longitudinal strain values compared with older adults, but the small differences of LS may be meaningless in clinical settings. The peak systolic CS showed significant differences except data of epicardium of basal and mid-ventricular l level between the young LVH and older LVH.

### 4.1 Usefulness of 2DSTE for evaluating myocardial deformation of young adults with hypertension

A number of studies on adults with hypertension have been published, but there is still controversy regarding the changes in LV performance.^[19,20]^ Moreover, these previous studies analyzed the myocardial function using the complete wall thickness without further distinction between different layers of the myocardium, and there are few investigations especially for young adults with hypertension. In China, young adults with hypertension have the lowest prevalence of blood pressure control compared to older adults. The important provider barriers to hypertension control with young adults including low rates of documented lifestyle counseling and significant delays prescribing initial antihypertension medication. Hypertension in young adulthood increases the risk of future cardiovascular events. To the best of our knowledge, this is the first study to demonstrate the deformation of layer-specific myocardium, endocardial, mid-ventricular, and epicardial layers in young adults with hypertension.

Among normal young adults, young NLVH, and young LVH, GLS and the peak systolic LS of mid-ventricular and apical level had significant differences. This finding might imply that the longitudinal strain is more susceptible to the young adults with hypertension than circumferential strain. The established concept that longitudinal contractile function as a more sensitive maker is prone to early pathological changes of the myocardium. Our findings in patients with hypertension and decreased tissue tracking values in the longitudinal fibers might be explained by the presence of regional subendocardial myocardial ischemia and increased perivascular and interstitial fibrosis, which were previously demonstrated in patients with hypertension.^[21–24]^ The subendocardial layer is susceptible to ischemia or fibrosis in patients with high blood pressure. Previous cardiac magnetic resonance (CMR) tagging study has suggested that decrease in circumferential radius of curvature during systole were more significant in endocardium than in epicardium, resulting higher endocardial strain, and in the early stage of hypertension (NLVH), the heart shows normal circumferential strain or even increased circumferential strain. In the NLVH stage, the circumferential strain was less. These results support the findings of our study. We conclude that the longitudinal strain can reflect systolic function very conveniently and accurately in adults with hypertension. The longitudinal function was very sensitive to early changes in adults with hypertension. However, in the LVH stage, the circumferential strain was attenuated, and we conclude that the systolic function was damaged.

In all the adults with hypertension, although young adults was associated with higher peak systolic longitudinal strain values compared with older adults, we did not observe significant age dependency with respect to either GLS or the peak systolic LS. No age dependency of longitudinal strain may have been due to either compensatory mechanisms or geometric changes, such as surface curvature. With regarding circumferential strain, the peak systolic CS showed significant differences except data of epicardium of basal and mid-ventricular level between the young LVH and older LVH. Our results suggest that circumferential strain may be more prone to the development of subtle changes in LV mechanics as aging process in adults with hypertension. When a heart is under a hemodynamic burden, the heart can use the Frank–Starling mechanism to augment muscle mass, and to recruit neurohormonal mechanisms to compensate. We conclude that in the early NLVH stage, longitudinal function was damaged first, in the LVH stage, both longitudinal function and circumferential function were damaged.

Hypertension is more prone to increase end-diastolic wall stress toward the endocardium, and endocardial fiber runs longitudinally. Therefore, the change in longitudinal strain always preceded LV hypertrophy in adults with hypertension, and there is no age dependency between the young patients and old patients. Circumferential function is affected mainly by the middle layers, that means the change in circumferential strain appear later than longitudinal strain. Circumferential strain always changes in the LV hypertrophy stage. The myocardial strain, contraction and diastole of young people are all different from the old people, so the circumferential strain between young patients and old patients with hypertension is different. If we use the normal ranges of old adults with hypertension to assess myocardial function of young adults with hypertension in clinical practice, myocardial damage may be not discovered. The normal range of myocardial layer-specific strain of young adults would enable more reliable diagnosis for myocardial damage in young adults with hypertension.

Although the hypertension has the same trend regarding layer-specific strain among age groups, there are significant differences between young adults with hypertension and the older ones, so we should know the normal reference values of layer-specific strain and the distinguishing feature of young adults with hypertension.

### 4.2 Study limitations

This study was characterized by several limitations. Firstly, we did not validate the accuracy of 2DSTE measurements against reference standard such as CMR in our study subjects. Secondly, the number of the hypertension patients is small, and long-term analysis with the larger samples is needed.

## 5 Conclusions

This study provides reference values for layer-specific strain in young adults with hypertension. This detailed strain analysis provides layer-oriented information to reveal the different characteristics of circumferential and longitudinal strain in young adults with hypertension. This systolic dysfunction could be detected conveniently and accurately with 2DSTE in young adults with hypertension.

## Author contributions

**Data curation:** Hong Zhou.

**Formal analysis:** Hong Zhou.

**Investigation:** Ning Wang, Yi Liang.

**Methodology:** Hong Zhou.

**Software:** Ning Wang, Xinxin Chen.

**Writing—original draft:** Liangjie Xu.

**Writing—review & editing:** Jinchuan Yan.
